# Expert Consensus on Key Attributes of Nurses in Resuscitation Teams: Findings From a Delphi Study

**DOI:** 10.1111/nicc.70506

**Published:** 2026-05-04

**Authors:** George Kipourgos, Nick Bakalis, Eleni Albani, Nikolaos Stefanopoulos, John Lakoumentas, Anastasios Tzenalis

**Affiliations:** ^1^ Surgical Cardiac Intensive Care Unit, Department of Cardiac Surgery, University of Patras Patras Greece; ^2^ Nursing Department University of Patras Patras Greece

**Keywords:** ALS, Delphi method, emergency nursing, expert consensus, in‐hospital cardiac arrest, non‐technical skills, nursing competencies, resuscitation team

## Abstract

**Background:**

In‐hospital cardiac arrest (IHCA) requires coordinated interdisciplinary action. Nurses are often first responders and essential members of resuscitation teams, yet the attributes that define their effectiveness remain unclear. Although team performance has been widely studied, few works have systematically examined nursing competencies in this context. This is the first Delphi‐based study in Greece defining key nursing attributes within in‐hospital resuscitation teams.

**Aim:**

To achieve expert consensus on the key attributes characterizing effective nursing participation in IHCA teams.

**Study Design:**

A two‐round Delphi study was conducted with experts in resuscitation and critical care. Round one involved thematic analysis of semi‐structured interviews. In round two, experts rated attributes on a 10‐point Likert scale. Consensus was defined as mean (*M*) > 8 and coefficient of variation (CV) < 20%. Descriptive statistics and Kendall's W assessed agreement across domains.

**Results:**

Thirty‐nine attributes were identified and grouped into seven domains: education, experience, physical condition, psychological resilience, technical skills and non‐technical skills. Thirty‐five attributes met the consensus criteria. Highest agreement was observed for ALS certification, stress resilience, closed‐loop communication, adaptability and teamwork. Strongest consensus emerged in non‐technical (*M* = 9.75, CV = 3.79%) and technical (*M* = 9.61, CV = 5.62%) domains.

**Conclusions:**

This study provides an evidence‐informed framework of competencies and personal qualities underpinning nurses' effectiveness in resuscitation teams, emphasizing both technical expertise and non‐technical skills—especially closed‐loop communication, composure and collaboration.

**Relevance to Clinical Practice:**

The framework supports clearer role delineation, structured competency development and enhanced team effectiveness in IHCA management.

## Impact Statements

- What is known about the topic
  ○ Effective in‐hospital resuscitation depends not only on technical proficiency but also on strong non‐technical skills, clear role allocation and coordinated teamwork.
  ○ Nurses frequently serve as first responders during in‐hospital cardiac arrest, yet the attributes that underpin their optimal performance are not clearly defined in the literature.
  ○ Existing training programmes (BLS, ILS and ALS) emphasize technical skills but do not comprehensively address the full range of competencies required in real clinical contexts.
  ○ The Delphi method is widely used to establish expert consensus in areas where empirical evidence is limited, heterogeneous or insufficient to guide clinical practice.
- What this paper adds
  ○ Identifies and prioritizes the essential attributes required for nurses' effective participation in in‐hospital resuscitation teams, based on expert consensus.
  ○ Provides a structured framework that can inform educational curricula and guide competency assessment processes in resuscitation‐related training.
  ○ Offers a practical foundation for selection criteria used to determine which nurses should staff resuscitation teams, supporting evidence‐based team composition.
  ○ Contributes to workforce development and organizational planning by highlighting key qualities relevant to emergency and resuscitation nursing practice.
  ○ Supports future research and the development of validated assessment tools aimed at examining how these attributes influence team performance and patient outcomes in cardiac arrest management.

## 1 Introduction

In‐hospital cardiac arrest (IHCA) remains a critical clinical event [1], characterized by high mortality rates and considerable variability in outcomes across healthcare systems [2, 3]. Despite continuous advancements in resuscitation guidelines and educational programmes [4, 5], the effectiveness and efficiency of resuscitation teams during IHCA events often depend on factors that extend beyond technical competence. Elements, such as team dynamics, clarity of roles, individual capabilities and context‐based decision‐making, critically influence the quality of care delivered in these high‐intensity situations [6, 7, 8].

### 1.1 Background

Within this context, the role of nurses as active members of resuscitation teams has attracted increasing research interest [9, 10]. Nurses are frequently among the first professionals to respond to cardiac arrest events, and their actions can significantly impact the chain of survival [11, 12]. However, the specific attributes that contribute to an effective nursing role during resuscitation remain insufficiently defined in the literature [13]. Evidence suggests that both clinical and non‐technical skills (NTSs)—such as leadership, communication and situational awareness—play a crucial role in team performance [14, 15, 16]. Nevertheless, there is no clear consensus regarding which attributes are most important or how they should be prioritized.

The growing emphasis on interprofessional collaboration and crisis resource management further underscores the need to define the role profiles of healthcare professionals within resuscitation teams [17, 18]. Although standardized educational programmes such as Advanced Life Support (ALS) provide a common technical framework [4], they do not adequately address the complex interplay of interpersonal skills, professional attitudes and real‐time clinical judgement—factors that frequently determine patient outcomes during cardiac arrest incidents [19, 20]. Moreover, variability in organizational models, staffing levels and professional experience complicates the adoption of unified frameworks for assessing nurses' competencies [21].

To address this gap, the Delphi technique offers a structured method for achieving expert consensus, particularly in areas where empirical evidence is limited or heterogeneous [22, 23]. By engaging professionals with validated expertise in resuscitation, the Delphi method facilitates the identification, refinement and confirmation of key parameters related to nurses' contributions to resuscitation teams [24]. It is especially suitable for the preliminary exploration of complex healthcare problems, enabling the systematic capture of expert knowledge, alignment of perspectives and development of shared conceptual frameworks [25].

### 1.2 Purpose of the Research

The aim of this study was to seek expert consensus on the essential attributes that define nurses' effective performance within in‐hospital resuscitation teams during cardiac arrest events.

## 2 Design and Methods

### 2.1 Design of Research

This study employed the Delphi technique to identify and prioritize the key attributes considered essential for the effective and high‐quality participation of nurses in in‐hospital resuscitation teams. The Delphi method is a systematic, iterative process for collecting and consolidating expert opinions with the aim of achieving consensus in areas where empirical evidence is limited or fragmented [24, 25, 26].

The methodological design consisted of two distinct rounds of data collection. The first round adopted a qualitative approach and involved semi‐structured interviews with selected experts in the field of resuscitation. Through thematic analysis, core dimensions and attributes deemed fundamental to the nursing role during resuscitation were identified. These findings formed the basis for the development of the second round.

The second round was conducted through an online platform (Typeform) and aimed to assess the degree of consensus among the experts regarding the relative importance of the attributes identified in the first round. Participants were asked to rate each attribute according to its significance for nurses' participation in resuscitation teams, using a 10‐point Likert scale [27]. Automatic anonymization of responses was implemented within the Typeform environment, ensuring confidentiality and enhancing methodological rigour.

The combined use of qualitative and quantitative exploration enabled an in‐depth understanding of the phenomenon under study and the formulation of an evidence‐informed framework grounded in expert consensus. The approach adopted can be characterized as a partially electronic Delphi, since the first round was conducted face‐to‐face, whereas the second round was implemented via a digital platform [28, 29].

### 2.2 Setting

**FIGURE 1:**
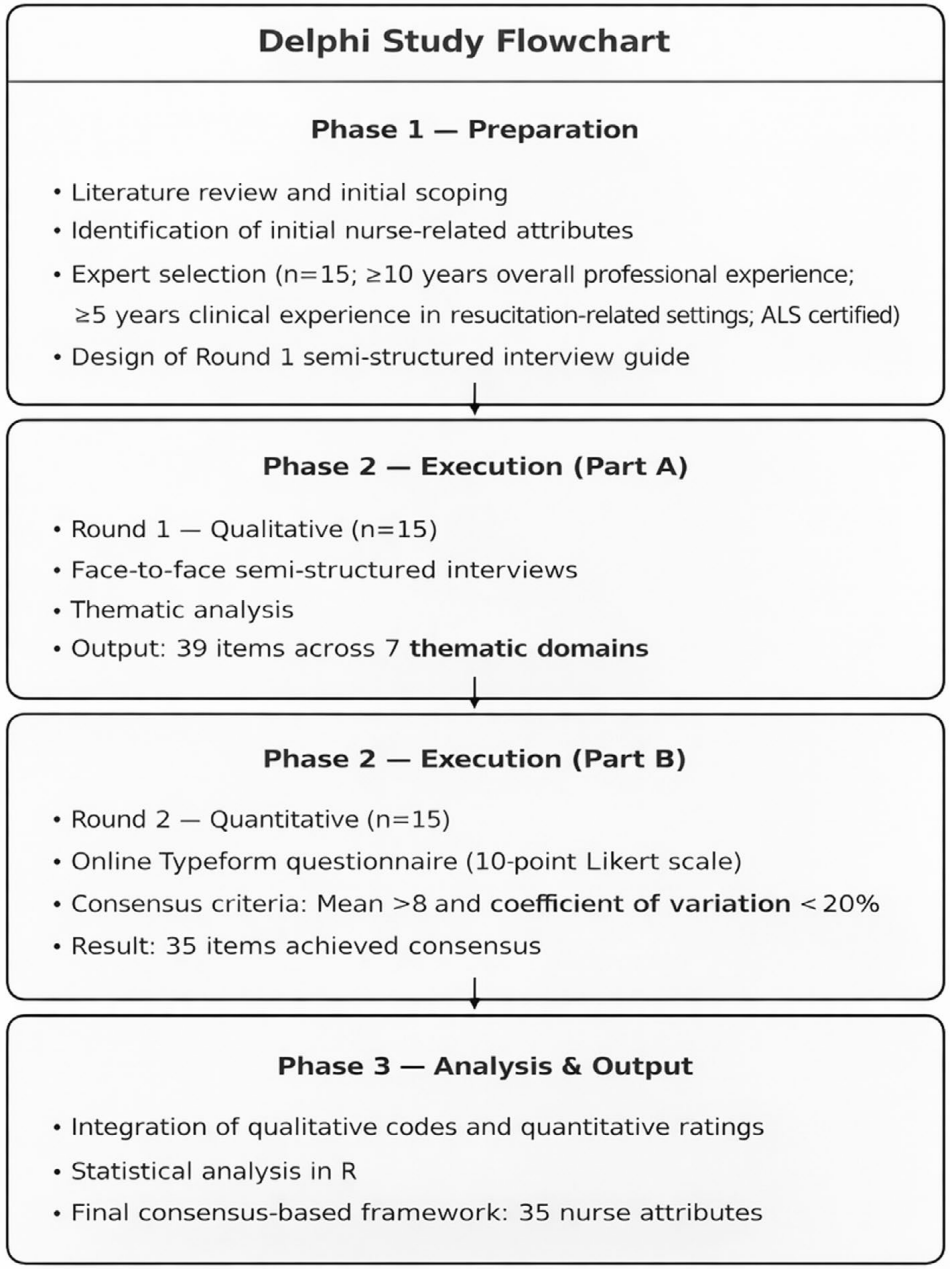
Delphi study flowchart. Overview of the three study phases: Preparation, execution (qualitative and quantitative rounds) and analysis/output, illustrating the methodological process and consensus development.

The implementation of the Delphi method in this study followed three distinct phases: the preparation phase, the conduction phase and the analysis phase (Figure 1). This sequential structure has been shown to ensure a systematic exploration of the phenomenon and the rigorous documentation of expert knowledge aimed at achieving consensus [30].

### 2.3 Delphi Preparation Phase—Participants

The preparation phase constituted the foundation of the Delphi process, as it defined the core parameters of the study and delineated its research scope. Initially, an extensive review of both international and Greek literature was conducted to capture existing perspectives regarding the role of nurses during IHCA and their participation in resuscitation teams. This review resulted in an initial pool of candidate attributes encompassing technical skills, NTSs, professional experience, physical capacity and psychological characteristics relevant to resuscitation performance.

This preliminary list of attributes was not intended to function as a predefined checklist or evaluative tool. Instead, it served as a conceptual framework to inform the development of the semi‐structured interview guide used in the first Delphi round, ensuring comprehensive thematic coverage while allowing experts to freely expand upon, refine or introduce additional attributes based on their clinical and professional experience.

Concurrently, the criteria for selecting the expert panel were established a priori. Selection was based on purposive sampling and predefined eligibility criteria, which included: (1) a minimum of 10 years of overall professional experience; (2) at least 5 years of clinical experience in resuscitation‐related settings (e.g., intensive care units [ICUs], emergency departments [EDs] or cardiac care units); and (3) valid certification in ALS. The application of these criteria aimed to ensure a high level of expertise, direct clinical exposure to IHCA events and familiarity with contemporary resuscitation standards.

The final panel consisted of 15 experts who agreed to participate and collectively represented a broad spectrum of knowledge and experience within resuscitation and critical care contexts.

Finally, the preliminary structure of the second‐round questionnaire was designed. The attributes identified during the first round were to be converted into clearly formulated statements (items), expressed in scientifically precise and neutral language to minimize response bias. For each statement, participants were asked to indicate its perceived importance using a 10‐point Likert scale (1 = Not at all important to 10 = Extremely important).

This phase ensured the validity of the study's theoretical framework, the clarity of its research objectives and the methodological consistency required for the successful application of the Delphi technique.

### 2.4 Data Collection

#### 2.4.1 Delphi Conduction Phase

The conduction phase involved the collection of expert knowledge through two distinct yet interrelated stages. The first stage adopted a qualitative approach, based on semi‐structured interviews with the 15 experts participating in the study. The interviews were conducted face‐to‐face, at a time and location agreed upon with each participant and were audio‐recorded with their consent. The transcribed content was analysed to identify the attributes considered critical for nurses' participation in resuscitation teams.

The development of the semi‐structured interview guide was informed by an extensive review of the international literature on nursing roles, competencies and team performance during IHCA. The full semi‐structured interview guide is provided in Appendix S1. This review resulted in an initial pool of candidate attributes, which was used as a conceptual framework to guide the interview process without constraining participants' responses. Interview questions were designed to balance open exploration with thematic prompts derived from this preliminary attribute list, allowing experts to elaborate freely, introduce additional attributes and contextualize their perspectives based on clinical experience.

The interview questions were designed to enable open exploration, allowing participants to freely express their perspectives on the essential qualifications, skills, attitudes and behaviours deemed important for effective nursing performance during resuscitation. During qualitative analysis, experts' responses were coded into thematic categories, which subsequently formed the basis for developing the second‐round questionnaire.

In the second stage, the process shifted towards quantitative orientation through the use of an electronic questionnaire hosted on the Typeform platform. The experts were invited to rate, on a 10‐point Likert scale, the degree of importance of the attributes identified in the first round. The electronic format ensured anonymity, accessibility, and standardized data collection, while facilitating participation without temporal or geographical constraints.

This hybrid approach—combining a face‐to‐face qualitative first round with an online quantitative second round—enhanced the methodological rigour of the study by providing both analytical depth and standardized assessment of expert opinions. Such a partially electronic Delphi design aligns with contemporary trends in nursing and medical research, promoting flexibility while maintaining scientific validity.

#### 2.4.2 Delphi Analysis Phase

The analysis phase involved the systematic processing of data collected during the two rounds of the Delphi procedure, aiming to generate evidence‐based conclusions and achieve valid consensus.

Data from the first round were analysed using thematic analysis under an interpretive framework. The transcribed interviews were examined line‐by‐line by two independent researchers who performed open coding of the data. The generated codes were then compared, grouped and categorized into broader thematic areas reflecting common patterns and meanings within participants' perspectives. The final set of themes was agreed upon through group discussion and methodological cross‐checking to ensure the reliability of the analysis and the representativeness of the data.

The thematic categories identified during the first round were converted into clear statements (items) for inclusion in the second‐round questionnaire. Consensus criteria were predefined as follows:

- Mean (*M*) > 8 (on a 1–10 scale).
- Coefficient of variation (CV) < 20%.

Attributes that met both criteria were considered to have achieved sufficient consensus among participants and were included in the final framework. Conversely, attributes showing wider dispersion or lower ratings were excluded from the final list.

The combined use of qualitative and quantitative analytical approaches strengthened the methodological validity of the study, ensuring both theoretical depth through expert interviews and empirical confirmation of the relative importance of each attribute through statistical convergence.

**FIGURE 2:**
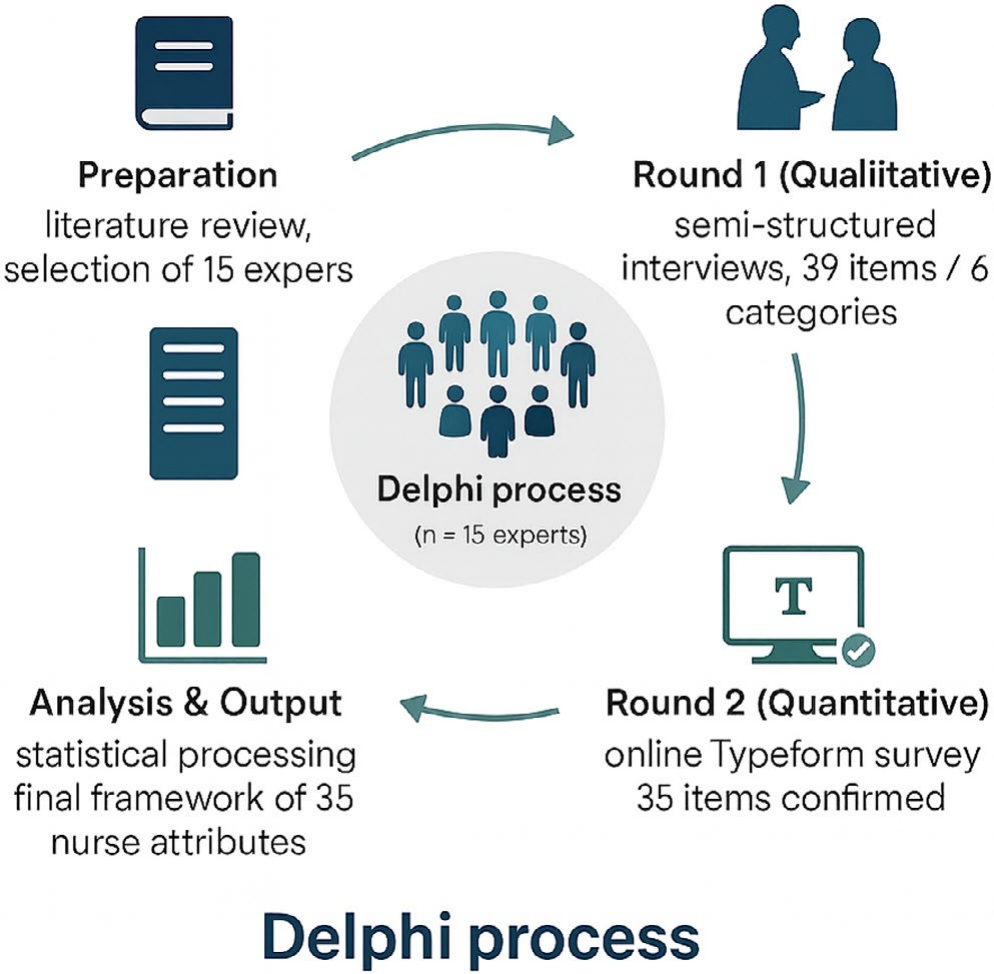
Delphi process. Schematic representation of the three phases of the Delphi process, from preparation and qualitative interviews to quantitative consensus and final analysis.

Content validity was established through the structured Delphi process, whereby attributes were generated, refined, and evaluated based on expert consensus (Figure 2).

### 2.5 Data Analysis

Regarding quantitative (numerical) methods, all the items of the questionnaire were assessed with descriptive statistical analysis. The mean (*M*) of all items valued from 1 to 10, the standard deviation (SD) and the CV, CV = 100 * SD/*M* (%), were computed. According to the rule of thumb [31], if the CV was higher than 20% a non‐consensus among the 15 experts was decided for the item. Identical statistics were estimated also for row‐wise sums of all the items for the total questionnaire, as well as for its components, such as education, experience, physical condition, mental condition, technical skills and NTSs. For the grouped items, Kendall's *W* index was also exploited to assess consensus among the 15 experts, and a corresponding *p*‐value was accordingly estimated, with a *p* < 0.05 denoting a statistically significant deviance from 0. An adequate consensus is considered when *W* > 30% [32]. Finally, the demographics of the 15 experts are provided as the mean ± SD for quantitative variables, while as the count (%) per categorical level for qualitative variables.

As a sensitivity analysis, median values and interquartile ranges (IQR) were additionally calculated for each individual item and for each component. Alternative consensus criteria (median ≥ 8 and IQR ≤ 2) were applied to assess the robustness of the findings to potential skewness or outliers.

Statistical analysis and visualization methods were performed in R (R version 4.1.2 ‘Bird Hippie’), along with the support of the RStudio IDE (RStudio ‘Ghost Orchid’ release).

The reporting of this study was guided by the Standards for Reporting Qualitative Research (SRQR) checklist (Table S2).

### 2.6 Ethical Considerations

The present study was conducted in full compliance with the principles of research ethics and deontology. Prior to commencement, ethical approval was obtained from the Research Ethics and Deontology Committee of the Nursing Department, University of Patras, Greece under protocol number 44531/14‐06‐2022.

All participants received detailed information regarding the purpose, content and methodology of the study, both verbally and through a written informed consent form. Participation was entirely voluntary, and participants retained the right to withdraw from the study at any stage without any consequences.

Particular emphasis was placed on maintaining anonymity and confidentiality. Participants' personal data were coded, and no information that could enable direct identification was retained. Interview transcripts were processed carefully in accordance with confidentiality protocols, and all data were securely stored in restricted‐access digital environments with enhanced protection measures.

Throughout the study, the ethical principles of the Declaration of Helsinki [33] and the General Data Protection Regulation (GDPR 2016/679) [34], were strictly followed, in accordance with Greek and European legislation. The research team demonstrated continuous sensitivity towards the participants' professional roles and expertise, ensuring an ethical balance between data collection and respect for their professional experience and autonomy.

## 3 Results

### 3.1 Characteristics of the Expert Panel

The expert panel that participated in this study consisted of 15 healthcare professionals with specialized knowledge and experience in resuscitation and emergency care. Selection was based on predefined criteria to ensure the scientific validity and thematic relevance of their contributions.

The sample included 15 experts with a mean age of 49.6 years (SD = 6.84). Ten participants (66.7%) were female and five (33.3%) were male. The majority were nurses (*n* = 11, 73.3%), whereas four participants (26.7%) were physicians. Regarding their professional setting, participants were primarily employed in ICUs, EDs, Cardiology Units, Anaesthesiology Departments and Hospital Education Offices. Additionally, several administrative positions were held (e.g., head nurses, sector managers, directors) or academic appointments. The detailed demographic and professional characteristics of the expert panel are presented in Table 1.

**TABLE 1:** Basic statistics of the demographic characteristics of the 15 experts that participated.

Demographic variable	Valid count	Descriptive statistic
Gender	15	
Male		5 (33.33%)
Female		10 (66.67%)
Age	15	49.60 ± 6.84
Occupation	15	
Doctor		4 (26.67%)
Nurse		11 (73.33%)
Experience	15	
10 years		1 (6.67%)
10–20 years		4 (26.67%)
> 20 years		10 (66.67%)
Graduate education	15	
Technological educational institute		9 (60%)
University education		6 (40%)
Post‐graduate education	15	
None		2 (13.33%)
Master		8 (53.33%)
Doctoral		4 (26.67%)
Post‐doctoral		1 (6.67%)

The composition of the panel highlights professionals with strong clinical and cognitive backgrounds, representing both frontline and educational or managerial domains of healthcare. This diversity provided a comprehensive overview of resuscitation and critical care practice in the Greek healthcare system.

Geographically, participants represented a wide range of institutions, including university hospitals, major regional healthcare centres, and academic or training institutions across Greece. All experts had direct or supervisory involvement in IHCA management.

The heterogeneity of the sample in terms of professional discipline, clinical experience and educational role enhanced the reliability of the data and facilitated the development of a well‐balanced and contextually grounded framework of nursing characteristics relevant to resuscitation team performance.

### 3.2 Findings of the First Round (Delphi 1)

During the first round of the study, semi‐structured interviews were conducted to gather expert opinions regarding the attributes considered critical for the effective participation of nurses in in‐hospital resuscitation teams. Through thematic analysis, a total of 39 distinct attributes were identified and categorized into seven thematic domains (Table 2):

- Education (e.g., theoretical knowledge, specialized training, certifications such as ALS).
- Age (e.g., demographic characteristic potentially associated with experience, physical capacity and response to high‐intensity clinical demands).
- Experience (e.g., years of clinical practice, exposure to resuscitation events).
- Physical condition (e.g., endurance, ability to perform rapid and demanding interventions).
- Mental condition (e.g., composure, stress resilience, anxiety management).
- Technical skills (e.g., execution of advanced clinical procedures, use of equipment).
- NTSs (e.g., communication, collaboration, leadership, teamwork).

**TABLE 2:** Distribution of Delphi Round 2 items by thematic category.

Thematic category	QID (questions)	Number of ITEMS
Education	Q1–Q8	8
Experience	Q9–Q13	5
Age group	Q14	1
Physical condition	Q15–Q16	2
Psychological condition	Q17–Q18	2
Technical skills	Q19–Q34	16
Non‐technical skills	Q35–Q39	5
Total	Q1–Q39	39

These thematic categories constitute a broad and comprehensive framework of attributes that reflect the multidimensional nature of the nursing role during resuscitation. The statements derived from the synthesis of expert opinions served as the basis for developing the second‐round questionnaire.

Although the first Delphi round was qualitative in nature, the distribution of attribute mentions across expert interviews demonstrated clear patterns of convergence. Several attributes were consistently mentioned by the majority of experts (e.g., more than two‐thirds of participants), while others emerged from a moderate proportion of interviews or were identified by a smaller number of experts. Importantly, attributes mentioned less frequently were retained for the second Delphi round in line with the exploratory aim of the qualitative phase and the inclusive principles of the Delphi methodology.

### 3.3 Consensus Findings of the Second Round (Delphi 2)

In the second round, all 15 experts evaluated the importance of the 39 attributes (Table 3) identified in the first round (response rate: 100%). No attrition occurred between rounds, and all analyses were conducted using complete‐case data.

**TABLE 3:** Delphi Round 2 statements (Q1–Q39).

QID	Statement
Q1	Education is a critical factor for nurses participating in resuscitation teams.
Q2	The higher the level of education, the more effective the nurse is as a team member in resuscitation.
Q3	Participation in ILS/ALS courses enhances the contribution of nurses in resuscitation teams.
Q4	Attendance of nursing specialties significantly improves effectiveness in resuscitation teams.
Q5	Holding a postgraduate or doctoral degree strengthens the role of nurses in resuscitation teams.
Q6	Attending educational programmes increases nurses' confidence during resuscitation team participation.
Q7	Attending educational programmes enhances collaboration levels with other team members.
Q8	Participation in certified courses (e.g., ILS, ALS) facilitates adaptation to updated resuscitation protocols.
Q9	Prior clinical experience (> 5 years) is a critical factor for effectiveness in resuscitation teams.
Q10	Work experience in critical care departments (ICU, ED, cardiology units, anaesthesiology) improves nurses' performance.
Q11	Clinical experience contributes to rapid recognition and response to cardiac arrest events.
Q12	Clinical experience enhances communication and teamwork within resuscitation teams.
Q13	Long‐term clinical experience increases the efficiency of nurses as resuscitation team members.
Q14	Younger age (< 45 years) is a positive factor for participation in resuscitation teams.
Q15	Good physical condition is a critical factor for effective participation in resuscitation teams.
Q16	A normal Body Mass Index (BMI) contributes positively to nurses' performance in resuscitation teams.
Q17	Psychological resilience is a key factor for effective participation in resuscitation teams.
Q18	Good physical and mental health enhances nurses' performance.
Q19	Assessment of consciousness level is an essential skill for nurses in resuscitation teams.
Q20	Airway control and basic airway opening techniques (e.g., jaw thrust, head tilt–chin lift) are essential skills.
Q21	Assisting in securing the airway (endotracheal intubation) is a critical skill.
Q22	Placement of oropharyngeal/nasopharyngeal airway is an essential skill.
Q23	Placement of a supraglottic device (e.g., laryngeal mask) is an essential skill.
Q24	Bag‐valve‐mask ventilation (ambu) is a critical skill.
Q25	Assessment of the respiratory system is a basic skill.
Q26	Assessment of the circulatory system is a basic skill.
Q27	Assessment of the central nervous system (e.g., Glasgow Coma Scale) is a basic skill.
Q28	Establishing intravenous and/or intraosseous access is an essential skill.
Q29	Drug administration is a basic skill.
Q30	Performing chest compressions is a basic skill.
Q31	Recognition of shockable and non‐shockable ECG rhythms is a critical skill.
Q32	Knowledge and recognition of reversible causes of cardiac arrest (4H's and 4T's) is an essential skill.
Q33	Recognition of ECG rhythms predicting return of spontaneous circulation (ROSC) is a critical skill.
Q34	Confirmation of ROSC is a basic skill.
Q35	Use of structured handover tools (e.g., SBAR) is an important skill for nurses in resuscitation teams.
Q36	Effective communication with other team members is a critical skill.
Q37	Effective collaboration with other team members is a critical skill.
Q38	Teamwork is an essential skill.
Q39	Frequent collaboration among resuscitation team members enhances performance and effectiveness.

Based on these criteria, 35 out of 39 attributes met the threshold for consensus and were included in the final framework. The remaining four were excluded due to limited agreement or higher response dispersion. The complete list of attributes with their respective consensus values is presented in Table 4.

**TABLE 4:** Numerical assessment of questionnaire items in the second Delphi round (10‐point Likert scale).

QID	Mean (*M*)	Standard deviation (SD)	Coefficient of variation (CV) %	Median	IQR
Q29	10.00	0.00	0.00	10.00	0.00
Q30	10.00	0.00	0.00	10.00	0.00
Q2	9.93	0.26	2.62	10.00	0.00
Q24	9.93	0.26	2.62	10.00	0.00
Q36	9.93	0.26	2.62	10.00	0.00
Q37	9.93	0.26	2.62	10.00	0.00
Q38	9.93	0.26	2.62	10.00	0.00
Q8	9.87	0.52	5.27	10.00	0.00
Q1	9.87	0.35	3.55	10.00	0.00
Q3	9.87	0.35	3.55	10.00	0.00
Q22	9.80	0.56	5.71	10.00	0.00
Q23	9.80	0.56	5.71	10.00	0.00
Q20	9.73	0.59	6.06	10.00	0.00
Q25	9.73	0.59	6.06	10.00	0.00
Q19	9.67	0.62	6.41	10.00	0.00
Q28	9.67	0.72	7.45	10.00	0.50
Q39	9.6	0.74	7.71	10.00	0.50
Q10	9.53	0.74	7.76	10.00	1.00
Q34	9.53	1.13	11.86	10.00	0.00
Q31	9.47	1.36	14.36	10.00	0.00
Q7	9.47	0.74	7.81	10.00	1.00
Q6	9.47	0.92	9.71	10.00	1.00
Q26	9.47	1.13	11.93	10.00	0.50
Q27	9.33	1.40	15.01	10.00	0.50
Q32	9.33	1.11	11.90	10.00	1.00
Q35	9.33	1.18	12.65	10.00	1.00
Q18	9.27	0.88	9.49	9.00	1.00
Q21	9.27	1.39	14.99	10.00	1.00
Q11	9.20	1.26	13.70	10.00	1.50
Q17	9.00	1.36	15.11	10.00	1.50
Q33	9.00	1.77	19.67	10.00	1.50
Q16	8.87	1.06	11.95	9.00	2.00
Q9	8.80	1.65	18.75	10.00	1.50
Q12	8.73	1.62	18.56	9.00	2.00
Q15	8.53	1.51	17.7	9.00	2.00
**Q13**	**8.47**	**2.47**	**29.16**	10.00	3.00
**Q14**	**8.13**	**1.68**	**20.66**	9.00	1.50
**Q4**	**8.00**	**1.65**	**20.62**	8.00	2.50
**Q5**	**6.47**	**2.42**	**37.40**	5.00	3.00

*Note:* QID denotes the item identifier. Descriptive statistics include mean (*M*), standard deviation (SD), coefficient of variation (CV), median and interquartile range (IQR). Primary consensus was determined using predefined criteria of *M* > 8 and CV < 20%. Median and IQR values are additionally reported as part of a sensitivity analysis to assess the robustness of the findings and were not used as primary criteria for consensus determination. Items are presented in descending order of *M*; items not meeting the consensus criteria are indicated in bold.

The attributes that did not meet the consensus criteria were related to:

- Completion of a nursing specialty programme.
- Possession of a master's or doctoral degree.
- The assumption that longer clinical experience necessarily leads to higher effectiveness as a member of the resuscitation team.
- The assumption that younger age (< 45 years) constitutes a critical positive factor for team participation.

The final list of 35 attributes reflects a comprehensive, expert‐validated framework of competencies, knowledge and behaviours deemed essential for nurses' participation in in‐hospital resuscitation teams. This framework may serve as a reference for both educational and clinical applications, aiming to enhance team effectiveness and interoperability in cardiopulmonary resuscitation (CPR) settings.

Analysis by thematic category revealed that the highest consensus levels were achieved for NTSs (*M* = 9.75, CV = 3.79%) and technical skills (*M* = 9.61, CV = 5.62%), indicating strong agreement among experts regarding their importance. In contrast, experience demonstrated the greatest variability (*M* = 8.95, CV = 14.53%), suggesting differing views on its relative influence. Overall, the questionnaire achieved an important level of acceptance (*M* = 9.33, CV = 5.04%), reflecting a robust degree of consensus across most evaluated attributes (Table S1).

All thematic categories and the total questionnaire were besides assessed regarding consensus via Kendall's *W* coefficient (and the corresponding statistical test). All of the estimated *W*‐values deviated in a statistically significant manner from 0 (all *p* < 0.05), denoting a consensus among experts. The total questionnaire was characterized by a *W* of 33.4%. The component that contained the maximal number of items, the education component, was characterized by a *W* of 56.9%. So, the hypothesis of a fair to moderate consensus was again confirmed.

## 4 Discussion

Through the systematic application of the Delphi method, this study identified a comprehensive set of attributes considered critical for the effective participation of nurses in in‐hospital resuscitation teams. The 35 validated attributes represent a consensus‐based framework that transcends technical proficiency, emphasizing the complex interplay between knowledge, skills and behavioural competencies required in high‐stakes clinical environments.

The findings align with previous international research underscoring the dual importance of technical skills and NTSs in resuscitation and emergency care contexts. Cooper et al. introduced the Team Emergency Assessment Measure (TEAM) as a standardized tool to evaluate teamwork, leadership and communication during resuscitation, demonstrating a strong association between these elements and clinical performance [15]. Similarly, Flin et al. emphasized that NTSs—such as situational awareness, decision‐making and leadership—are as decisive as technical competence in determining team effectiveness during critical incidents [16]. Rosen et al. (United States) further highlighted teamwork and shared mental models as fundamental contributors to patient safety and high‐quality care delivery within healthcare teams [17].

Experts identified a set of attributes—including ALS certification and adaptability, which received the highest mean ratings, as well as psychological resilience and closed‐loop communication behaviours, which demonstrated strong consensus despite not being among the highest‐rated items—as essential components of effective participation in resuscitation teams. Similar findings have been reported by Liaw et al. (Singapore), who demonstrated that structured interprofessional simulation training significantly improved nurses' communication confidence and response accuracy in deteriorating patient scenarios [9]. Likewise, Fernandez et al. identified teamwork and leadership as key factors shaping the efficiency and decision‐making quality of emergency care teams [14].

Another noteworthy aspect of the findings concerns the interpretation of ‘formal education’. Although attributes referring specifically to postgraduate or doctoral degrees did not reach consensus, the statement indicating that a higher overall level of education is associated with greater effectiveness as a member of the resuscitation team received one of the highest ratings and the lowest dispersion among all items. This pattern suggests that experts strongly value education as a foundational determinant of clinical reasoning, situational awareness and preparedness during resuscitation events.

The apparent distinction may reflect a pragmatic differentiation made by the panel between general educational level—which is perceived as essential for effective team participation—and the incremental contribution of research‐oriented academic degrees, which may not be viewed as directly enhancing real‐time performance in high‐acuity resuscitation settings. This interpretation aligns with evidence from studies conducted in Europe and North America indicating that team effectiveness in emergency care relies more heavily on applied knowledge, shared practices and team‐based training than on formal academic titles alone [21, 35].

It is also noteworthy that experts strongly agreed that more than 5 years of clinical experience in critical care settings represents a key characteristic for effective participation in resuscitation teams. However, they did not support the assumption that age is a determining factor in performance, as no elevated level of consensus was observed regarding either younger age (< 45 years) or the notion that longer clinical experience automatically translates into higher team efficiency.

This finding highlights the distinction between quantitative experience (years in practice) and qualitative experience (repeated exposure to real cardiac arrest events and continuous training). Comparable results were reported by Hunziker et al. (Switzerland), who found that team performance during cardiac arrest did not depend primarily on age or years of experience, but rather on regular participation in simulation‐based scenarios and clear role allocation [8]. Similarly, Couper and Perkins emphasized that structured debriefing following resuscitation events is a stronger predictor of performance improvement than overall professional tenure [7]. Fernandez Castelao et al. (Germany) also demonstrated that participation in Crisis Resource Management (CRM) programmes significantly enhances team performance regardless of participants' age or seniority [6], whereas Marshall and Flanagan observed that decision‐making and communication under pressure are skills developed through deliberate and repeated practice rather than mere longevity in service [18].

Overall, these observations support the concept of adaptive expertise, suggesting that clinical effectiveness in complex, high‐intensity settings relies on cognitive flexibility, reflective learning and continuous exposure to realistic clinical challenges rather than on years of professional experience alone [36].

Although psychological resilience and composure under pressure were not among the highest‐ranked or most tightly clustered attributes in the quantitative findings, their endorsement by the expert panel highlights their relevance as underlying components of broader NTSs. In the present study, resilience was primarily captured through a single item, which may partly explain its comparatively lower mean rating and greater dispersion. Nevertheless, experts appeared to conceptualize psychological resilience as a foundational capability that supports effective communication, decision‐making and performance under stress during resuscitation events.

Consistent with this interpretation, Al Thobaity et al. (Saudi Arabia) and Jubinville et al. (Canada) reported that resilience, adaptability and clear role allocation substantially influence nurses' performance in emergencies, particularly in resource‐limited environments [19, 20]. This aligns with broader evidence indicating that simulation‐based team training significantly improves human factor skills relevant to crisis performance [37].

From an educational perspective, the present framework provides actionable insights for curriculum developers and policymakers. Incorporating these attributes into structured programmes such as Basic Life Support (BLS), Immediate Life Support (ILS) and ALS, as well as into simulation‐based education, can enhance the realism, relevance and transferability of learning outcomes. This approach aligns with the European Resuscitation Council (ERC) Guidelines 2025, which emphasize human factors, team behaviour and cognitive load management as key learning objectives [4].

The present framework should be interpreted as a consensus‐based conceptual foundation rather than a ready‐to‐use assessment or selection tool. While the identified attributes provide valuable direction for resuscitation education, team training and curriculum development, further psychometric validation is required before the framework can be operationalized into standardized measurement instruments or used for formal performance appraisal.

In this context, the findings may inform the design of educational curricula, simulation‐based training programmes and future research protocols aimed at evaluating nursing performance during resuscitation. Subsequent studies should focus on scale development, reliability and validity testing and examination of associations between the proposed attributes and objective clinical outcomes.

### 4.1 Limitations

Despite the methodological rigour and expert validation of its findings, this study is not without limitations.

The assessment of attribute importance relied solely on subjective expert judgements, without the possibility of correlating these with objective performance indicators (e.g., return of spontaneous circulation [ROSC], response time, survival to discharge). Therefore, empirical validation of the identified characteristics in real clinical contexts is warranted.

While the Delphi method is ideal for achieving consensus, it does not ensure absolute truth but rather represents the collectively accepted opinion of a specific expert group within a defined context. The results should therefore be interpreted as reflecting consensus rather than universal agreement.

Despite these limitations, this study provides a strong theoretical and practical foundation that can serve as a starting point for further research and for applications in nursing education, performance assessment and clinical practice.

A further limitation of this study relates to the geographical composition of the expert panel. All participating experts were recruited from Greece, reflecting the structure, organization and professional roles within the Greek healthcare system. While the Delphi methodology aims to capture expert consensus rather than population representativeness, contextual factors such as national training pathways, scope of nursing practice and resuscitation team organization may influence the prioritization of specific attributes. Consequently, caution is warranted when transferring these findings directly to other healthcare systems or regions. Future studies involving multinational expert panels could further explore the generalizability and cross‐cultural applicability of the proposed framework.

## 5 Implications and Field of Application

The findings of this study should be interpreted as a consensus‐based conceptual framework rather than a ready‐to‐use assessment or decision‐making tool. Developed through expert agreement, the proposed set of characteristics provides structured insight into key domains relevant to nurses' participation in in‐hospital resuscitation teams, while requiring further empirical validation prior to operational implementation.

From an educational perspective, the identified characteristics may inform the design of learning objectives within BLS, ILS and ALS training programmes as well as within emergency nursing curricula and simulation‐based education. In this context, the framework can support the alignment of educational content with expert‐defined priorities, particularly with regard to NTSs and team‐based performance.

At an organizational and administrative level, the framework may guide discussions around role expectations, professional development pathways, and the structuring of induction or mentorship programmes for nurses involved in resuscitation care. However, its use for formal personnel selection, performance appraisal, or credentialing should be approached with caution until psychometric testing and contextual validation have been completed.

From a research perspective, the present framework offers a foundation for the development of psychometrically validated measurement instruments and for future quantitative or experimental studies. It may also inform the design of research protocols aimed at evaluating nursing performance during resuscitation. Such work should focus on scale development, reliability and validity testing, as well as on the examination of associations between the proposed attributes and objective clinical outcomes, including team performance metrics and patient‐centred outcomes.

Overall, the framework represents an important step towards a more structured and theory‐informed understanding of the nursing role in resuscitation, while underscoring the need for continued validation and refinement before widespread practical application.

## 6 Conclusion

Through the systematic application of the Delphi method, this study identified a set of 35 attributes considered by experienced professionals to be critical for nurses' effective participation in in‐hospital CPR teams. These attributes encompass a wide spectrum of skills, knowledge and behaviours, integrating both technical competencies and NTSs such as communication, teamwork, leadership and emotional resilience.

The resulting framework can serve as a practical guide for strengthening educational processes, conducting targeted staff assessments and enhancing the selection and onboarding procedures for nurses joining specialized resuscitation teams. Moreover, it provides a reliable foundation for developing new research instruments and for further exploring the relationship between these attributes and objective clinical performance and outcome indicators.

In a high‐intensity and complex field such as resuscitation, documenting the qualitative dimensions of the nursing role is an essential prerequisite for ensuring safety, effectiveness and interoperability within resuscitation teams. The contribution of this study lies in defining such a framework—grounded in the knowledge, experience and consensus of the specialized clinical community.

## Funding

The authors have nothing to report.

## Ethics Statement

The present study was conducted in full compliance with the principles of research ethics and deontology. Prior to commencement, ethical approval was obtained from the Research Ethics and Deontology Committee of the Nursing Department, University of Patras, Greece under protocol number 44531/14‐06‐2025. All procedures followed the Declaration of Helsinki and GDPR (EU Regulation 2016/679).

## Consent

The authors have nothing to report.

## Conflicts of Interest

The authors declare no conflicts of interest.

## Supporting information


**Appendix S1:** Semi‐structured Interview guide.

**Table S1:** Descriptive statistics of Delphi Round 2 items by component. The analysis shows higher consensus in non‐technical and technical skills, while experience exhibited greater variability among experts.
**Table S2:** SRQR (Standards for Reporting Qualitative Research) checklist.

## Data Availability

The data that support the findings of this study are available from the corresponding author upon reasonable request.

## References

[1] J. Soar , B. W. Böttiger , P. Carli , et al., “European Resuscitation Council Guidelines 2025 Adult Advanced Life Support,” Resuscitation 215, no. Suppl 1 (2025): 110769, 10.1016/J.RESUSCITATION.2025.110769. 41117572

[2] S. Girotra , B. K. Nallamothu , J. A. Spertus , Y. Li , H. M. Krumholz , and P. S. Chan , “Trends in Survival After In‐Hospital Cardiac Arrest,” New England Journal of Medicine 367 (2012): 1912–1920, 10.1056/NEJMOA1109148. 23150959 PMC3517894

[3] L. W. Andersen , M. J. Holmberg , K. M. Berg , M. W. Donnino , and A. Granfeldt , “In‐Hospital Cardiac Arrest: A Review,” Journal of the American Medical Association 321 (2019): 1200–1210, 10.1001/JAMA.2019.1696. 30912843 PMC6482460

[4] S. Nabecker , T. de Raad , C. Abelairas‐Gomez , et al., “European Resuscitation Council Guidelines 2025 Education for Resuscitation,” Resuscitation 215 (2025): 110739, 10.1016/J.resuscitation.2025.110739. 41117567

[5] J. G. Wigginton , S. Agarwal , J. A. Bartos , et al., “Part 9: Adult Advanced Life Support: 2025 American Heart Association Guidelines for Cardiopulmonary Resuscitation and Emergency Cardiovascular Care,” Circulation 152 (2025): S538–S577, 10.1161/CIR.0000000000001376. 41122884

[6] E. Fernandez Castelao , S. G. Russo , S. Cremer , et al., “Positive Impact of Crisis Resource Management Training on No‐Flow Time and Team Member Verbalisations During Simulated Cardiopulmonary Resuscitation: A Randomised Controlled Trial,” Resuscitation 82 (2011): 1338–1343, 10.1016/j.resuscitation.2011.05.009. 21664757

[7] K. Couper and G. D. Perkins , “Debriefing After Resuscitation,” Current Opinion in Critical Care 19 (2013): 188–194, 10.1097/MCC.0B013E32835F58AA. 23426138

[8] S. Hunziker , A. C. Johansson , F. Tschan , et al., “Teamwork and Leadership in Cardiopulmonary Resuscitation,” Journal of the American College of Cardiology 57 (2011): 2381–2388, 10.1016/j.jacc.2011.03.017. 21658557

[9] S. Y. Liaw , W. T. Zhou , T. C. Lau , C. Siau , and S. W. Chan , “An Interprofessional Communication Training Using Simulation to Enhance Safe Care for a Deteriorating Patient,” Nurse Education Today 34 (2014): 259–264, 10.1016/j.nedt.2013.02.019. 23518067

[10] G. E. Ougli , B. Boukatta , A. E. Bouazzaoui , et al., “Impact of Simulation on the Development of Nursing Students' Competence in Adult Cardiopulmonary Resuscitation,” Cureus 16 (2024): e72722, 10.7759/CUREUS.72722. 39618661 PMC11606625

[11] P. S. Chan , S. L. Krein , F. Tang , et al., “Resuscitation Practices Associated With Survival After In‐Hospital Cardiac Arrest: A Nationwide Survey,” Journal of the American Medical Association Cardiology 1 (2016): 189–197, 10.1001/JAMACARDIO.2016.0073. 27437890 PMC5745254

[12] L. J. Morrison , R. W. Neumar , J. L. Zimmerman , et al., “Strategies for Improving Survival After In‐Hospital Cardiac Arrest in the United States: 2013 Consensus Recommendations: A Consensus Statement From the American Heart Association,” Circulation 127 (2013): 1538–1563, 10.1161/CIR.0B013E31828B2770. 23479672

[13] A. Sukarwan , Y. Peristiowati , and A. D. Ellina , “The Role of Emergency Nurses in Cardiopulmonary Resuscitation (CPR) of Cardiac Arrest Patients: A Literature Review,” ENDLESS: International Journal of Future Studies 4 (2021): 148–158, 10.54783/ENDLESS.V4I1.53.

[14] R. Fernandez , S. W. J. Kozlowski , M. J. Shapiro , and E. Salas , “Toward a Definition of Teamwork in Emergency Medicine,” Academic Emergency Medicine 15 (2008): 1104–1112, 10.1111/J.1553-2712.2008.00250.X. 18828831

[15] S. Cooper , R. Cant , J. Porter , et al., “Rating Medical Emergency Teamwork Performance: Development of the Team Emergency Assessment Measure (TEAM),” Resuscitation 81 (2010): 446–452, 10.1016/j.resuscitation.2009.11.027. 20117874

[16] R. Flin , P. O'Connor , and M. Crichton , Safety at the Sharp End: A Guide to Non‐Technical Skills (CRC Press, 2017), 10.1201/9781315607467.

[17] M. A. Rosen , D. DiazGranados , A. S. Dietz , et al., “Teamwork in Healthcare: Key Discoveries Enabling Safer, High‐Quality Care,” American Psychologist 73 (2018): 433–450, 10.1037/AMP0000298. 29792459 PMC6361117

[18] S. D. Marshall and B. Flanagan , “Simulation‐Based Education for Building Clinical Teams,” Journal of Emergencies, Trauma, and Shock 3 (2010): 360–368, 10.4103/0974-2700.70750. 21063559 PMC2966569

[19] A. Al Thobaity , “Overcoming Challenges in Nursing Disaster Preparedness and Response: An Umbrella Review,” BMC Nursing 23 (2024): 1–11, 10.1186/S12912-024-02226-Y. 39143575 PMC11323674

[20] M. Jubinville , E. N. Tchouaket , and C. Longpré , “Scoping Review Protocol Examining Charge Nurse Skills: Requirement for the Development of Training,” BMJ Open 13 (2023): e067307, 10.1136/BMJOPEN-2022-067307. PMC9950913 36822804

[21] M. Buljac‐Samardzic , K. D. Doekhie , and J. D. H. Van Wijngaarden , “Interventions to Improve Team Effectiveness Within Health Care: A Systematic Review of the Past Decade,” Human Resources for Health 18 (2020): 2, 10.1186/S12960-019-0411-3. 31915007 PMC6950792

[22] S. Keeney , F. Hasson , and H. Mckenna , The Delphi Technique in Nursing and Health Research (2010), Wiley, 10.1002/9781444392029.

[23] F. Hasson , S. Keeney , and H. McKenna , “Research Guidelines for the Delphi Survey Technique,” Journal of Advanced Nursing 32 (2000): 1008–1015, 10.1046/J.1365-2648.2000.T01-1-01567.X. 11095242

[24] I. R. Diamond , R. C. Grant , B. M. Feldman , et al., “Defining Consensus: A Systematic Review Recommends Methodologic Criteria for Reporting of Delphi Studies,” Journal of Clinical Epidemiology 67 (2014): 401–409, 10.1016/j.jclinepi.2013.12.002. 24581294

[25] R. Boulkedid , H. Abdoul , M. Loustau , O. Sibony , and C. Alberti , “Using and Reporting the Delphi Method for Selecting Healthcare Quality Indicators: A Systematic Review,” PLoS One 6 (2011): e20476, 10.1371/JOURNAL.PONE.0020476. 21694759 PMC3111406

[26] N. Dalkey and O. Helmer , “An Experimental Application of the DELPHI Method to the Use of Experts,” Management Science 9 (1963): 458–467, 10.1287/MNSC.9.3.458.

[27] “A Technique for the Measurement of Attitudes: Rensis Likert,” n.d., accessed August 11, 2025, https://archive.org/details/likert‐1932.

[28] M. Niederberger and J. Spranger , “Delphi Technique in Health Sciences: A Map,” Frontiers in Public Health 8 (2020): 457, 10.3389/FPUBH.2020.00457. 33072683 PMC7536299

[29] W. Vernon and W. Vernon , “The Delphi Technique: A Review,” International Journal of Therapy and Rehabilitation 16 (2009): 69–76, 10.12968/IJTR.2009.16.2.38892.

[30] D. Beiderbeck , N. Frevel , H. A. von der Gracht , S. L. Schmidt , and V. M. Schweitzer , “Preparing, Conducting, and Analyzing Delphi Surveys: Cross‐Disciplinary Practices, New Directions, and Advancements,” MethodsX 8 (2021): 101401, 10.1016/J.MEX.2021.101401. 34430297 PMC8374446

[31] C. G. Fraser , “Inherent Biological Variation and Reference Values,” Clinical Chemistry and Laboratory Medicine 42 (2004): 758–764, 10.1515/CCLM.2004.128. 15327011

[32] J. R. Landis and G. G. Koch , “The Measurement of Observer Agreement for Categorical Data,” Biometrics 33 (1977): 159, 10.2307/2529310. 843571

[33] World Medical Association , “World Medical Association Declaration of Helsinki: Ethical Principles for Medical Research Involving Human Subjects,” Journal of the American Medical Association 310 (2013): 2191–2194, 10.1001/JAMA.2013.281053. 24141714

[34] “Regulation – 2016/679 – EN – GDPR – EUR‐Lex,” n.d., accessed October 9, 2025, https://eur‐lex.europa.eu/eli/reg/2016/679/oj.

[35] R. F. Friedlaender , E. M. Gubert , C. M. B. Fernandes , R. G. Mello , and I. C. M. M. Coelho , “Evaluation of Acquisition and Retention of Non‐Technical Skills of Residents Submitted to Interprofessional Simulation‐Based Training in Pediatric Cardiopulmonary Resuscitation,” Jornal de Pediatria 101 (2025): 394–399, 10.1016/j.jped.2024.12.003. 39894449 PMC12039372

[36] D. Schwartz , J. Bransford , and D. Sears , “Efficiency and Innovation in Transfer,” in Transfer of Learning From a Modern Multidisciplinary Perspective, ed. J. Mestre (Information Age Publishing, 2005), 1–51, https://www.scirp.org/reference/referencespapers?referenceid=538270.

[37] L. Abildgren , M. Lebahn‐Hadidi , C. B. Mogensen , et al., “The Effectiveness of Improving Healthcare Teams' Human Factor Skills Using Simulation‐Based Training: A Systematic Review,” Advances in Simulation 7 (2022): 12, 10.1186/S41077-022-00207-2. 35526061 PMC9077986

