# Supplementary material for: Expert Consensus on Key Attributes of Nurses in Resuscitation Teams: Findings From a Delphi Study
Source: Nurs Crit Care. 2026 May 4;31:e70506. doi: 10.1111/nicc.70506 (PMC13139511; doi:10.1111/nicc.70506)
Supplement: Supplementary file 1 — Appendix S1: Semi‐structured Interview guide. [file NICC-31-0-s002.docx]

**Appendix S1. Semi-Structured Interview Guide**

1. *Based on your experience, which attributes do you consider essential for nurses participating in in-hospital resuscitation teams?*
2. *Which technical skills do you believe are critical for effective nursing performance during cardiac arrest events?*
3. *What non-technical skills (e.g., communication, leadership, teamwork) are particularly important in resuscitation settings?*
4. *How do experience, training, and certification influence a nurse’s effectiveness within a resuscitation team?*
5. *What psychological or emotional characteristics do you consider important for nurses during high-stress resuscitation situations?*
6. *Are there any attributes or competencies you believe are undervalued or insufficiently addressed in current resuscitation training programs?*
7. *Do you consider certain attributes to be context-dependent (e.g., ICU vs ward, night shifts, staffing conditions)? Please elaborate.*
