# Supplementary material for: Expert Consensus on Key Attributes of Nurses in Resuscitation Teams: Findings From a Delphi Study
Source: Nurs Crit Care. 2026 May 4;31:e70506. doi: 10.1111/nicc.70506 (PMC13139511; doi:10.1111/nicc.70506)
Supplement: Supplementary file 2 — Table S1: Descriptive statistics of Delphi Round 2 items by component. The analysis shows higher consensus in non‐technical and technical skills, while experience exhibited greater variability among experts. Table S2: SRQR (Standards for Reporting Qualitative Research) checklist. [file NICC-31-0-s001.docx]

# Supplementary Tables

| **Component** | **Mean (M)** | **Standard Deviation (SD)** | **Coefficient of Variation (CV) %** | **Median** | **IQR** | **Kendall's W** | **P-VALUE** |
| --- | --- | --- | --- | --- | --- | --- | --- |
| Education | 9.12 | 0.56 | 6.14 | 10 | 1 | 0,569 | <0,001 |
| Experience | 8.95 | 1.30 | 14.53 | 10 | 2 | 0,140 | 0,079 |
| Physical condition | 8.70 | 1.01 | 11.61 | 9 | 2 | 0,145 | 0,069 |
| Psychological condition | 9.13 | 1.08 | 11.83 | 10 | 1 | 0,151 | 0,059 |
| Technical skills | 9.61 | 0.54 | 5.62 | 10 | 0 | 0,139 | 0,008 |
| Non-technical skills | 9.75 | 0.37 | 3.79 | 10 | 0 | 0,177 | 0,031 |
| Overall questionnaire | 9.33 | 0.47 | 5.04 | 10 | 1 | 0,334 | <0,001 |

# Table S1. Descriptive statistics of Delphi Round 2 items by component. The analysis shows higher consensus in non-technical and technical skills, while experience exhibited greater variability among experts.

# Table S2. SRQR (Standards for Reporting Qualitative Research) Checklist

| **SRQR Item** | **Description** | **Where addressed in manuscript** |
| --- | --- | --- |
| **1** | Title | Title page |
| **2** | Abstract | Abstract |
| **3** | Problem formulation | Introduction |
| **4** | Purpose or research question | Introduction |
| **5** | Qualitative approach and research paradigm | Methods (Design and Methods) |
| **6** | Researcher characteristics and reflexivity | Not applicable (Delphi expert consensus study) |
| **7** | Context | Methods (Setting and Study Design) |
| **8** | Sampling strategy | Methods (2.3 Delphi Preparation Phase – Participants) |
| **9** | Ethical issues pertaining to human subjects | Methods (Ethical considerations) |
| **10** | Data collection methods | Methods (2.4 Delphi Conduction Phase) |
| **11** | Data collection instruments and technologies | Methods (Semi-structured interview guide; Typeform questionnaire) |
| **12** | Units of study | Methods (2.3 Participants; n = 15 experts) |
| **13** | Data processing | Methods (2.4.1 Qualitative analysis; coding and categorization) |
| **14** | Data analysis | Methods (2.5 Data Analysis) |
| **15** | Techniques to enhance trustworthiness | Methods (Delphi design, anonymity, iterative rounds) |
| **16** | Synthesis and interpretation | Results |
| **17** | Links to empirical data | Results (Tables and figures) |
| **18** | Integration with prior work | Discussion |
| **19** | Limitations | Discussion (Limitations) |
| **20** | Conclusions | Discussion and Conclusions |
| **21** | Implications | Discussion (Educational and clinical implications) |
| **22** | Funding | Funding statement |
| **23** | Conflicts of interest | Conflicts of interest statement |
